# Uniform arrays of gold nanoelectrodes with tuneable recess depth

**DOI:** 10.3762/bjnano.12.72

**Published:** 2021-08-30

**Authors:** Elena O Gordeeva, Ilya V Roslyakov, Alexey P Leontiev, Alexey A Klimenko, Kirill S Napolskii

**Affiliations:** 1 Lomonosov Moscow State University, Leninskie Gory, Moscow 199991, Russia; 2 Kurnakov Institute of General and Inorganic Chemistry RAS, Leninsky av., Moscow 119991, Russia; 3 Institute of Nanotechnology of Microelectronics RAS, Leninsky av., Moscow 115487, Russia; 4 Moscow Institute of Physics and Technology, Institutskiy per., Dolgoprudny 141701, Russia

**Keywords:** anodic alumina, gold, nanoelectrode array, recessed electrode, templated electrodeposition

## Abstract

Nanoelectrode arrays are much in demand in electroanalytical chemistry, electrocatalysis, and bioelectrochemistry. One of the promising approaches for the preparation of such systems is templated electrodeposition. In the present study, porous anodic alumina templates are used to prepare Au nanoelectrode arrays. Multistage electrodeposition is proposed for the formation of recessed electrodes with the ability to tune the distance between the surface of the porous template and the top surface of the nanoelectrodes. A set of complementary techniques, including chronoamperometry, coulometry, and scanning electron microscopy, are used to characterize the nanoelectrode arrays. The number of active nanoelectrodes is experimentally measured. The pathways to further improve the recessed nanoelectrode arrays based on anodic alumina templates are discussed.

## Introduction

A nanoelectrode array (NEA) is a set of regularly arranged isolated metal electrodes with a small radius (usually less than 50 nm), which is comparable or less than the thickness of the electric double layer [1–2]. The main advantages of such systems include high mass-transfer rates under steady-state diffusion, diminished electric double layer capacitance, and short response times [1–5]. Nowadays, Pt and Au NEAs find a wide range of applications in electroanalytical chemistry, electrocatalysis, bioelectrochemistry, and kinetic measurements [6–10].

Several strategies have been applied for the fabrication of NEAs. Modern instrumental techniques (such as direct-writing using electron beam lithography [11–12] or ion beam milling [13–14]) are limited by the ensemble area and expensive in mass production, but allow one to precisely tune the parameters of an array (a geometry of individual electrodes and the distance between them) over a wide range. An alternative approach includes bulk electrode structuring by deposition or etching techniques using self-assembled arrays of colloidal nanoparticles [15], liquid crystals [16], or track-etched membranes [17–19] as template or mask, respectively. Among porous templates, anodic aluminium oxide (AAO) allows one to create NEAs with a narrow distribution of geometrical parameters (diameter of electrodes and distance between them) that can be tuned over a wide range [20–21].

The first attempt to prepare recessed nanoelectrodes based on AAO templates was made in 2002 [9]. The authors used commercially available porous AAO films with a highly irregular structure to prepare the Au nanowell electrodes with 600 nm depth by templated electrodeposition. It is worth noting that the percentage of nanoelectrodes in the array that are involved in electrochemical reactions has not been discussed in detail. At the same time, only a minor part of nanowires is known to grow through the whole thickness of a template when potentiostatic electrodeposition is used [22–25]. Not only electrodeposition conditions but also thickness and structural defects in the AAO template influence the completeness of the template filling [26–27].

There are several requirements for structure and properties of NEAs, that includes, (1) mechanical stability and the ability to control the geometric parameters of nanoelectrodes, (2) chemical stability in electrolyte solutions, (3) recess uniformity for the electrodes in the array and the ability to control the recess depth, and (4) a predictable number of nanoelectrodes in electrical contact with the current collector. In the present study, the first two points are fulfilled by using AAO as a template and Au as the material of the working part of the nanoelectrodes. To address the third issue, a new design of the Au NEAs with a multi-layered structure has been suggested. The fourth requirement has been proved experimentally using electrochemical methods and scanning electron microscopy.

## Results and Discussion

**Figure 1:**
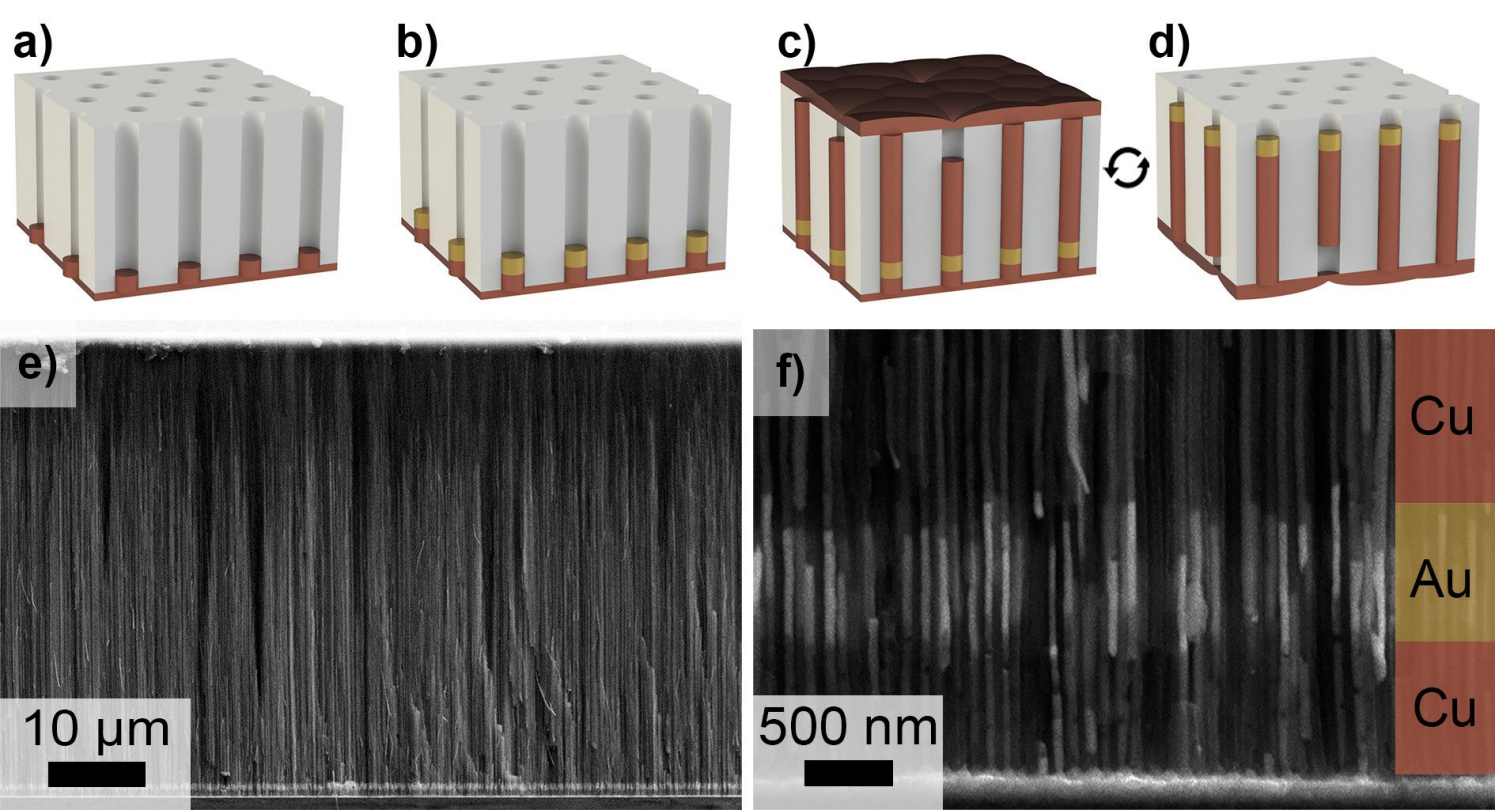
The flow chart of the NEA preparation: (a) deposition of Cu current collector and subsequent electrodeposition of the first Cu segment; (b) electrodeposition of the second Au segment; (c) electrodeposition of the third Cu segment until the formation of a continuous Cu layer on the surface of AAO template; (d) turning the AAO template upside down with subsequent selective dissolution of the current collector and the first Cu segment. Cross-sectional SEM images of AAO/Cu/Au/Cu nanocomposite, schematically shown in panel (c), illustrating the full thickness of the template (e) and the bottom part with two short segments of Cu and Au (f).

The first stage of NEA preparation (Figure 1a) was the formation of a short (up to 1 µm) Cu segment, which governed the recess of NEAs relative to the surface of the AAO template. At the second stage (Figure 1b), a short (up to several micrometers) Au segment was electrodeposited above the Cu segment. Further, these Au segments will serve as an electroactive surface in the proposed NEAs. At the third stage (Figure 1c), Cu was grown until the nanowires reached the AAO top surface and a continuous metal layer was formed on it. This Cu layer served as current collector during operation of the Au NEAs. Finally, the initial Cu current collector and the first Cu segment were selectively etched away after turning the AAO template upside down (Figure 1d).

### Segment 1 – copper

Optimization of the deposition potential (*E*_d_) for the formation of the first Cu segment was performed in the range from −0.1 to −0.5 V. In the case of more negative potentials, an intensive hydrogen evolution manifests itself by the decrease in the current efficiency (η). It ranged from 98% to 100% for *E*_d_ values from −0.1 to −0.3 V, whereas, the η value at *E*_d_ = −0.4 V decreased below 97% (Table 1). The electrodeposition at potentials above −0.1 V leads to a significant decrease in the Cu growth rate as can be seen from the dependence of the average current density (*j*_aver_) and the Cu growth rate on the deposition potential (Table 1).

**Table 1:** Parameters for the first Cu segment electrodeposition.

Deposition potential (*E*_d_), V	Current efficiency (η), %	Average current density (*j*_aver_), mA·cm^−2^	Growth rate, µm·h^−1^	Cu segment length (*L*_Cu1_), µm	Standard deviation of Cu segment length (Δ*L*), %

−0.1	99.7	4.5	93	9.42 ± 0.70	7.5
−0.2	98.3	13.5	199	6.74 ± 0.15	2.5
−0.3	99.3	15.1	314	9.04 ± 0.43	4.8
−0.4	96.8	18.5	322	7.48 ± 0.47	6.3
−0.5	—	22.3	344	7.06 ± 0.49	6.9

To determine the Cu electrodeposition conditions leading to the highest length uniformity, the first segment with much longer length than the supposed recess was formed. According to cross-sectional SEM images of the Cu/AAO nanocomposites, the length of the segments obtained at different *E*_d_ varied from 6.7 to 9.5 µm (Table 1). The electrodeposition of Cu at *E*_d_ = −0.2 V allowed one to obtain arrays of Cu segments with the most uniform length, and thus, this value was chosen as an optimal *E*_d_ for further experiments. According to the scheme of the NEA fabrication (Figure 1), the first Cu segment’s length (*L*_Cu1_) determines the recess of NEAs relative to the template surface. In order to enhance a response time of sensing material inside AAO, *L*_Cu1_ was decreased to 0.64 ± 0.09 µm (Figure 1f). It is worth noting that a decrease in *L*_Cu1_ value leads to a significant reduction of the absolute length deviation.

### Segment 2 – gold

The high chemical stability of Au makes it an intrinsic material for electrochemical sensors and has motivated the choice of this metal for the second segment. The low concentration of Au(I) electroactive species in the electrolyte results in a low current density (*j*_aver_ ≈ 0.6 mA·cm^−2^ for *E*_d_ = −1.0 V) and a low metal growth rate of 3.5 µm·h^−1^. As a consequence, complete pore filling in the used AAO template requires ca. 14 h. Such a long-term Au electrodeposition from acidic electrolyte with pH < 5 leads to the degradation of the AAO template, characterized by a low chemical stability in the as-prepared amorphous state [28–29]. Thus, the proposed design and strategy for the fabrication of the Au NEAs include the formation of a short Au segment (0.57 ± 0.06 µm) with the subsequent filling of the rest of the pore by Cu (Figure 1e,f). The use of a bright Cu plating solution with a high concentration of Cu(II) allows one to decrease the electrodeposition time down to 30 min and obtain a continuous metal layer on the AAO top surface.

### Segment 3 – copper

At this step, a key quality parameter is the percentage of pores filled by the metals completely from the bottom to the top of the AAO template. Only nanowires in this type of pores can take part in an electrochemical reaction during the operation of the NEAs because only these nanowires are electrically connected both to the bottom and the top continuous Cu layers (Figure 1c). According to [27], the decrease in electrodeposition overpotential, which is accompanied by the decrease in the diffusion current contribution, leads to an increase in the number of nanowires that reach the AAO top surface. As a consequence, a narrower *E*_d_ range from −0.3 to −0.1 V was chosen for an optimization of the electrodeposition conditions for the third segment of Cu.

**Figure 2:**
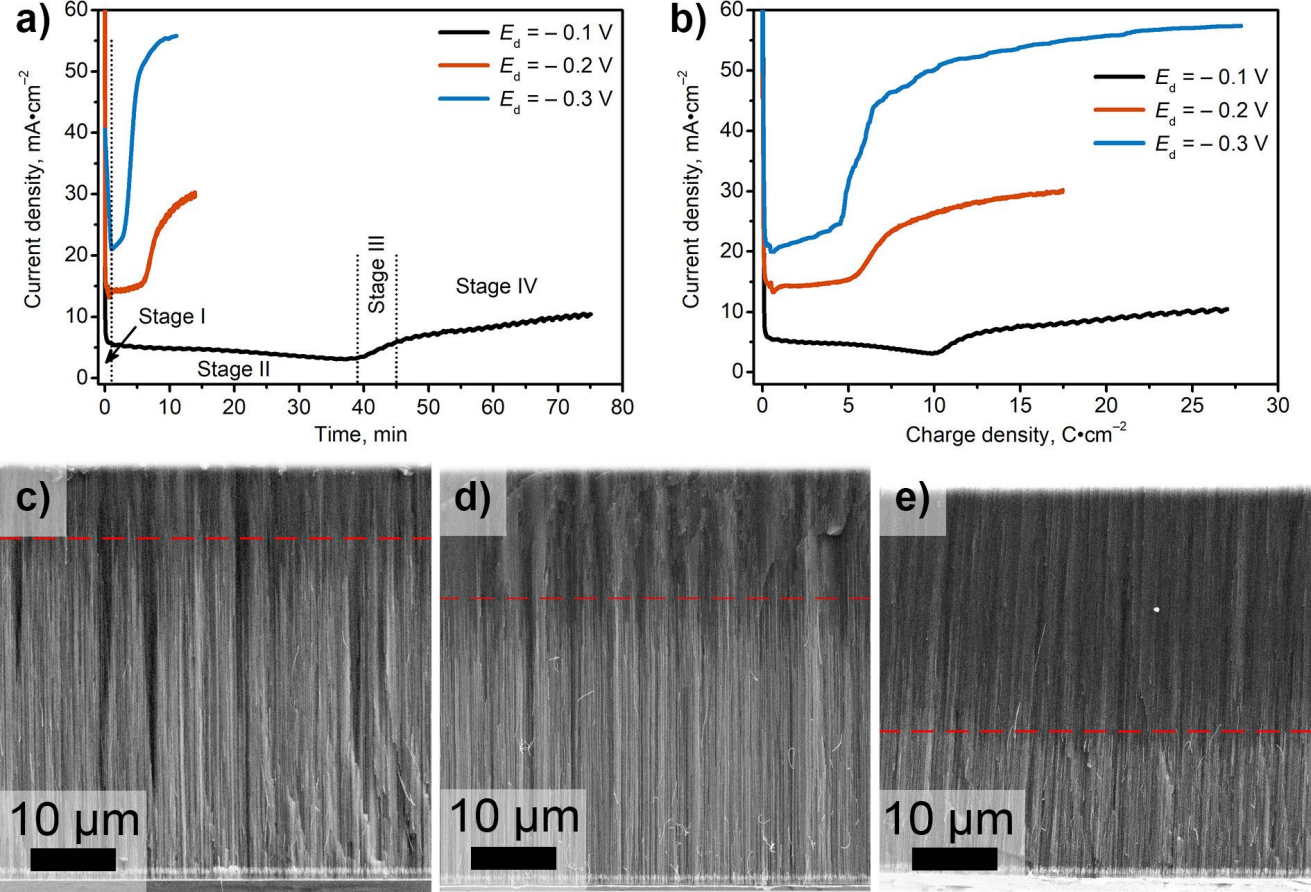
Electrochemical responses recorded during the electrodeposition of the third Cu segment: (a) *j*–*t* curves and (b) *j*–*q* curves. Characteristic stages of the templated electrodeposition are indicated in panel (a) on the example of the current transient recorded at *E*_d_ = −0.1 V. Cross-sectional SEM images of the AAO/Cu/Au/Cu nanocomposites obtained at different deposition potentials of the third Cu segment: (c) *E*_d_ = −0.1 V, (d) *E*_d_ = −0.2 V, and (e) *E*_d_ = −0.3 V. Dashed red lines indicate the position of the main growth front of the Cu nanowires.

The current density as a function of the time, recorded at different *E*_d_, is demonstrated in Figure 2a. At stage I, a sharp drop in current density is associated with the charging of the electric double layer, a decrease in the concentration of electroactive species near the electrode surface, and an increase in the diffusion layer thickness. Stage II corresponds to the growth of Cu segments inside the AAO at near constant current density. At stage III, some of the nanowires reach the AAO surface and Cu starts to grow at the external surface of the template, which manifests itself by a growth of current density. Finally, the current density stabilizes at a near constant value, indicating the growth of a continuous metal film (stage IV).

The charge density, which passes before the metal nanowires reach the AAO surface (*q*_exp_), is strongly affected by *E*_d_ (Figure 2b). An increase in overpotential leads to a decrease in the average filling the AAO template with metal. According to Faraday’s law, the electric charge density required for homogeneous filling of all pores (*q*_calc_) is ca. 9 C·cm^−2^ (Table 2). The average filling factor (*q*_exp_/*q*_calc_) of the AAO template is about 100% in the case of *E*_d_ = −0.1 V and exhibits a drastic drop as the overpotential rises. A similar behavior was observed earlier for templated electrodeposition of Ni [24] and Cu [27] in AAO templates.

**Table 2:** Completeness of the AAO template filling during the electrodeposition of the third Cu segment.

Deposition potential (*E*_d_), V	Calculated charge density (*q*_calc_), C·cm^−2^	Experimental charge density (*q*_exp_), C·cm^−2^	*q*_exp_/*q*_calc_, %	Cu segment length (*L*_Cu3_), µm	Ratio of Cu segment length to AAO thickness (*L*_Cu3_/*H*), %

−0.1	9.05	10.0	108	39.5 ± 1.9	81
−0.2	9.20	5.0	53	33.8 ± 1.6	69
−0.3	9.07	4.5	48	17.4 ± 1.4	36

Cross-sectional SEM images were used for the estimation of the position of the main growth front of nanowires (see dashed red lines in Figure 2c–e) at the final stage of the templated electrodeposition. It can be seen that the main growth front is below the top surface of the AAO template. Such structure results from the inhomogeneity of the growth rates in neighboring pores: when a limited number of segments reach the AAO surface, the growth of other ones stops due to a screening effect. At *E*_d_ = −0.1 V, many segments reach the template surface, and the growth front is about 10 µm from the top surface of the AAO template. The average filling factor, calculated as a ratio between the position of the main growth front (*L*_Cu3_) and the AAO thickness (*H*), is equal to 81% (Table 2). At *E*_d_ = −0.2 V and −0.3 V, the length of the nanowires is smaller, and the average filling factors are 69% and 36%, respectively.

Thus, according to the current–time transients and SEM data, the maximum average filling factor of the AAO template is observed when the deposition potential of the third Cu segments is equal to −0.1 V. At this potential, the greatest number of Cu segments having electrical connection both with the bottom and the top continuous Cu layers are formed.

### Formation and characterization of NEAs

After all the steps described above, the obtained NEAs consisted of the following components: the Cu layer sputtered on the AAO bottom side, short Cu, short Au, and long Cu segments inside the template, and the continuous electrodeposited Cu layer that covers the AAO top surface (Figure 1c). At the final step of NEA formation, the Au segments were opened by selective dissolution of the initially sputtered current collector as well as the short segment of Cu after turning the AAO template upside down (Figure 1d). As a result, an array of 28 nm diameter gold electrodes (electrode-to-electrode distance is equal to 101 nm) with 0.6 µm recess depth was obtained.

**Figure 3:**
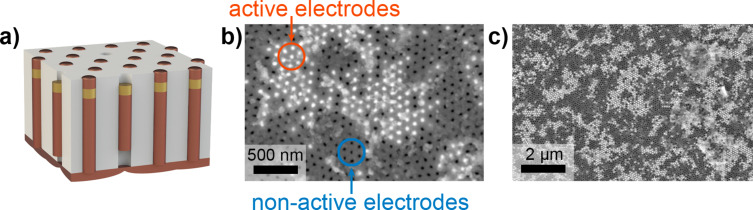
An NEA after Cu re-deposition onto recessed Au electrodes: 3D schematic drawing (a) and top-view SEM images recorded at high (b) and low (c) magnification.

Electrochemical characterization of the prepared NEAs was performed. To estimate the number of electrodes with electrical contact, Cu was re-deposited on the surface of the Au segments (Figure 3a). The experiment was carried out at *E*_d_ = −0.1 V and limited by the electrical charge density, which is equal to the corresponding value in the case of electrodeposition of the first Cu segment.

SEM images of the AAO surface after Cu re-deposition are shown in Figure 3b,c. The Au nanoelectrodes having electrical contact with the current collector manifest themselves as white dots, indicating that Cu reaches the template surface. Contrary, the black dots correspond to the pores containing recessed Au electrodes without re-deposited Cu. According to a statistical analysis of the SEM images, (45 ± 15)% of the nanoelectrodes are active (i.e., have electrical contact with the Cu current collector).

The main reason why some of the electrodes have no electric contact with the current collector is a variation in the growth rate of the third Cu segments due to the contribution of diffusion current. Thus, an increase in the fraction of active nanoelectrodes could be achieved under kinetic control of electrodeposition process, which requires lower overpotentials [24,26] and/or lower electrolyte temperatures [30–31]. Moreover, thin AAO templates with highly ordered porous structure (and, as a consequence, small quantity of the branched pores) can be used [27,32–33]. One more useful approach that can be applied for the increase in the number of contacting nanowires is planarization of the nanocomposite surface using mechanical polishing or ion etching and subsequent re-deposition of the current collector.

It is worth noting that a great number of Au electrodes within the array is extremely important for a practical application of NEAs under the kinetic control of a target electrochemical process. In this case, the process current strongly depends on the electro-active area of the working electrode. Contrary, in the case of diffusion control of the target process, the large fraction of active electrodes is not a key requirement for NEAs. Due to the small distance between electrodes, overlapping of the individual diffusion layers results in the planar diffusion to the NEA surface. Thus, a decrease in the density of active electrodes could be more appropriate because of the decrease in electric double layer charging current and, as a consequence, the growth of the signal-to-noise ratio. A controlled decrease in the active nanoelectrodes in the array can be achieved by modification of the AAO structure and artificial pore branching [34].

## Conclusion

Templated electrodeposition was successfully applied for the fabrication of recessed Au NEAs. The proposed preparation protocol includes a three-stage electrodeposition of Cu/Au/Cu segments into the pores of an anodic alumina template with the subsequent selective dissolution of the sputtered Cu current collector and the first Cu segment. The reported design of NEAs allows one to tune the recess of Au nanoelectrodes relative to the AAO surface with high precision.

NEAs with Au electrodes of 28 nm in diameter, an electrode-to-electrode distance of 101 nm, and a recess relative to the template surface of 640 ± 90 nm were obtained. The average filling factor of AAO templates exceeded 80% under optimal electrodeposition conditions. For the first time, the number of the active recessed nanoelectrodes was quantified. It was shown that 45 ± 15% of electrodes have electrical contact with the current collector.

The obtained recessed NEAs are prospective for creating electrochemical sensors, in which the template sterically stabilizes the sensing material. It is necessary when the active material is chemically and/or mechanically unstable on the surface of plate electrodes [35–36]. Also, the proposed electrodes can be used to study the electrochemical properties of conjugated oligomers, which can be selectively immobilized in the recess with suitable depth and size [37]. The first sacrificial Cu segment allows one to achieve a highly homogeneous recess of the Au electrodes. Moreover, a relatively short Au working segment with the remaining part of the electrode of Cu makes the technology economically attractive.

## Experimental

Au NEAs were prepared by a multi-step templated electrodeposition technique. To begin with, high-purity Al foils (99.99%, 100 µm thick, GOST 25905−83) were electrochemically polished to a mirror finish as described elsewhere [38]. Formation of AAO templates was performed by a two-stage anodizing of Al in 0.3 M H_2_C_2_O_4_ electrolyte at a constant voltage of 40 V. The temperature of the electrolyte was kept constant at 20 °C. The anodizing duration was controlled coulometrically using a thickness-to-charge density ratio of 500 nm·cm^2^·C^−1^ [39]. At the first stage, a sacrificial oxide layer with a thickness of 10 µm was formed and then selectively etched away in a solution containing 0.5 M H_3_PO_4_ and 0.2 M CrO_3_ at a temperature of 70 °C for 10 min. The resulting Al foil with an ordered lattice of concaves on the surface was re-anodized under the same conditions until an oxide layer with a thickness of 50 µm was formed. After the second anodizing stage, the remaining Al was selectively dissolved in 10 vol % Br_2_ solution in CH_3_OH. Then, the barrier layer of the AAO template was removed by chemical etching in 3.5 M H_3_PO_4_ with electrochemical detection of the pore opening moment as described elsewhere [40]. The prepared AAO templates were 49.0 ± 0.1 µm in thickness (*H*) and possessed a periodic 2D hexagonal arrangement of pores of the diameter *D*_p_ = 28 ± 6 nm with an interpore distance *D*_int_ = 100.9 ± 0.7 nm. The calculated porosity was 7%, and the pore density was 1.1 × 10^10^ cm^−2^.

To form a current collector, a 150 nm thick Cu layer was deposited by magnetron sputtering onto the bottom side of the AAO template. Electrodeposition of metals into channels of the AAO template was performed in a three-electrode cell with an electrodeposition area of 0.2 cm^2^ and a volume of 50 mL at room temperature in a potentiostatic mode. A platinum ring served as counter electrode. A saturated (KCl) Ag/AgCl electrode was used as a reference electrode (*E*_Ag/AgCl_ − *E*_SHE_ = 0.197 V). All potentials hereinafter are given versus Ag/AgCl reference electrode. The length of the segments was controlled coulometrically. The Cu segments were electrodeposited using the commercial electrolyte Ecomet MB-16 [41]. Optimization of the deposition potential (*E*_d_) in the range from −0.1 to −0.5 V was performed. The current efficiency was determined using gravimetric analysis of Cu layers on the plain Au electrodes. Au was electrodeposited from the commercial electrolyte Ecomet 04-ZG [42] using *E*_d_ equal to −1.0 V. Preliminary nucleation pulses of −0.5 V and −1.2 V for 0.1 s were applied in the cases of Cu and Au electrodeposition, respectively.

The initial Cu current collector and the first Cu segment were selectively dissolved in a solution containing 0.3 M H_2_SO_4_ and 0.9 М H_2_O_2_ for 30 min at room temperature.

Characterization of the morphology of the AAO templates and NEAs was carried out using scanning electron microscopes (SEM) Carl Zeiss NVision 40 and Leo Supra 50VP.
